# Testing the Social Bubble Hypothesis on the Early Dynamics of a Scientific Project: The FET Flagship Candidate FuturICT (2010–2013)

**DOI:** 10.3390/e23101279

**Published:** 2021-09-29

**Authors:** Monika Gisler, Didier Sornette

**Affiliations:** 1 Department of Management, Technology and Economics, ETH Zürich, Scheuchzerstrasse 7, 8092 Zürich, Switzerland; 2 Swiss Finance Institute, c/o University of Geneva, 40 blvd. Du Pont d’Arve, CH 1211 Geneva, Switzerland

**Keywords:** social bubbles, innovation, risk-aversion, cost-benefit, ethnography, EU flagship, herding, group behavior

## Abstract

We present an analysis of a large emerging scientific project in the light provided by the *social bubbles hypothesis* (SBH) that we have introduced in earlier papers. The SBH claims that, during an innovation boom or technological revolution, strong social interactions between enthusiastic supporters weave a network of reinforcing feedbacks that leads to widespread endorsement and extraordinary commitment, beyond what would be rationalized by a standard cost–benefit analysis. By probing the (Future and Emerging Technologies) FET Flagship candidate FuturICT project, as it developed in 2010–2013, we aimed at better understanding how a favorable climate was engineered, allowing the dynamics and risk-taking behaviors to evolve. We document that significant risk-taking was indeed clearly found—especially during workshops and meetings, for instance, in the form of the time allocation of participants, who seemed not to mind their precious time being given to the project and who exhibited many signs of enthusiasm. In this sense, the FuturICT project qualifies as a social bubble in the making when considered at the group level. In contrast, risk-perception at the individual level remained high and not everyone involved shared the exuberance cultivated by the promoters of FuturICT. As a consequence, those not unified under the umbrella of the core vision built niches for themselves that were stimulating enough to stay with the project, but not on a basis of blind over-optimism. Our detailed field study shows that, when considering individuals in isolation, the characteristics associated with a social bubble can vary significantly in the presence of other factors besides exaggerated risk-taking.

## 1. Introduction

Human societies are complex dynamic systems that evolve as a result of the interplay between technological changes, socio-political transformations resulting from wars, and revolutions and structural organization, together with dynamics of debt and inequality. In this context, innovation plays a central role, as shown by transformations over the last 250 years marked by a series of “industrial revolutions” (IR) that revolutionised civilisation: IR1 (1750 to 1850): coal, steel, steam and railroads; IR2 (1870 to 1930): electricity, internal combustion engine, cars, running water, indoor toilets, telephone, wireless telegraphy and radio, movies, petroleum, and chemicals; IR3 (1960 to 2000): electronics, computers, the web, the Internet, and mobile phones; IR4 (on-going, from 2000 to the uncharted future): the progressive fusion of the physical, digital and biological worlds with cloud computing, information storage, the Internet of things, the Blockchain technology revolution, artificial intelligence, intelligent robots, self-driving cars, genomics and gene editing, and neuro-technological developments.

Even though the literature on innovation is vast, what actually drives innovation is still a hotly discussed matter. A particularly important question is how to create an environment that provides the necessary conditions for innovation to flourish. This question is particularly important nowadays, when monetary and fiscal policies have shown their limitations in rebooting the economies of developed countries after the Great Recession that followed the great financial crisis of 2008. Ultimately, innovation is the engine of productivity growth, which itself is the only way to increase wealth per inhabitant. In a series of articles [1,2,3], the two present authors have articulated the “social bubble” hypothesis to explain the mechanism at the origin of previous waves of technological innovation and to study the conditions that are favorable for future innovations.

The social bubble hypothesis proposes that, during an innovation boom or technological revolution, strong social interactions between enthusiastic supporters of an idea/concept/project weave a network based on positive feedback, leading to widespread endorsement and extraordinary commitment by those involved in the respective project beyond what would be rationalized by a standard cost–benefit analysis [4]. It does not cast any value system, however, notwithstanding the use of the term “bubble,” which is often associated with a negative outcome. Rather, it identifies the types of dynamics that shape scientific or technological endeavors. In other words, we suggest that major projects can (and often do) proceed via a social bubble mechanism, which turns out to play a positive role in fostering their development. In order to push this hypothesis further, we present a case study analyzing a major scientific project in its early phase.

The term “bubble” is borrowed from finance, where it refers to the behavior of speculators buying assets, knowing that their prices are far above any reasonable estimation of a “fundamental value,” expecting that they will rise further before they may ultimately crash. Within the “rational expectation” model of such bubbles, it remains rational for an investor to ride the bubble because the occurrence of the crash is not certain and there is a non-zero probability for the bubble to end without a crisis. Other models argue that bubbles must involve an “irrational expectation” component, associated with a belief that the person to whom you are offering an asset will be willing to buy the asset and will not see the crash coming [5,6,7,8,9]. The imperative of an enduring crisis following a bubble has been questioned occasionally, as it has been contended that bubbles can also have positive effects on the economy and society in the long run by creating productive systems (infrastructure, technology, etc. [10,11]).

Concepts such as Schumpeter’s famous “creative destruction” and the “technological economic paradigm shift” of the social economist Carlota Perez [11,12], based on Schumpeter, target questions similar to ours. Perez is interested in bubbles as antecedents of “techno-economic paradigm shifts.” She describes the dynamics of a bubble as a rapid growth of new technologies funded by financial capital that is derived from investment and speculation, leading to a speculative frenzy and eventually to a crash. This does not, however, necessarily end in a blow; rather, Perez claims, its sequel is characterized by its restructuring, turning into steady growth as the new technology is rolled out, eventually leading to maturity and exhaustion, characterized by the installation of a new technical paradigm. Consequently, she designates bubbles as inevitable. Daniel Gross [13], on the other hand, studying well-known bubble episodes in history, points to the circumstance in which investors’ money is used to build infrastructures that cannot possibly repay their upfront costs, but end up being beneficial for companies and consumers in the long run. To take a recent case, most investors in “dotcoms” lost—but their money built the software and infrastructure that runs today’s Internet.

Complementing the macro-perspective framed above, we engage in a micro-perspective of such social bubble episodes by studying their detailed dynamics. We hypothesize that a social bubble is a phenomenon that develops in a social system during a technological or scientific project. In our analyses of the US-Apollo Program [1] and the Human Genome Project [3], we delineated different phases of the process:

- strong support for a specific idea/project by different interest groups, based on highly inflated expectations, establishing exuberance around it;
- credit creation via public and private investment;
- proliferation of ventures of all kinds and accelerated price growth of corresponding firms trading on organized stock markets;
- saturation of the idea, exhaustion of the interest and the flow of capital towards the project, and program stagnation or termination, at the end.

During these social bubbles, both the program and the project were nucleated and engineered by special interest groups whose optimistic claims about future outcomes (similar to the features of over-optimism; e.g., [14,15]) attracted more and more people to join the project. Such inflated expectations led to an even higher risk appetite by people focusing solely on future returns, forgetting the associated risks. Large public investment was the consequence, fostering at some point the proliferation of private ventures (start-ups, venture capital) eventually investing heavily into the projects. This resonates with an analysis by Mariana Mazzucato [16], wherein she stresses the public sector’s centrality to risk-taking and radical growth fostering technological change. She builds a very different picture of the state from that envisaged by present economic policy, which denies it any leading role in innovation and production.

In order to add more case studies to the social bubble hypothesis and to analyze its phases in depth, we opted to look into a project in the making: a FET Flagship candidate called FuturICT (previously at http://www.futurict.eu (accessed on 25 September 2021) (discontinued) and recycled to the present since 2017 as the FuturICT 2.0 project funded by the European Commission’s FLAG-ERA program. https://futurict2.eu (accessed on 25 September 2021)). By probing the FuturICT project as it developed in 2010–2013, we aimed at better understanding how a favorable climate was being engineered that allowed any interested party to join in. This was a unique opportunity to study the dynamics and risk-taking behaviors associated with an innovation process in its developmental phase.

FuturICT was an EU Coordination Action project that aspired to become one of Europe’s leading research projects on Future and Emerging Technologies (FET). The overall goal of FuturICT was to understand techno-socio-economic world systems by building a platform where simulating and visualizing large amounts of social data would enable policy-makers and entrepreneurs, as well as society at large, to acquire new information to facilitate decision making and hence prevent risks in the long-run (http://www.futurict.eu (accessed on 6 December 2015)).

One could raise the question of whether our study can be compared to those of financial bubbles, which constitute the inspiration for the social bubble hypothesis, that involve investors who are prone to herding [17,18,19,20,21,22]. Can herding develop among scientists? There is no doubt that science also functions according to waves of fashions, where certain ideas or fields emerge, find strong and sometimes enthusiastic support, and then fade. For the physical sciences, one could name the field of chaos in the early 1980s, which was supposed to provide generic tools for describing the world’s complexity, or the concept of self-organized criticality in the 1990s, allegedly providing a unifying theory of the universe. Here we adopt the position that science is just one system among others within society, and hence is anything but different to any other system.

**Figure 1:**
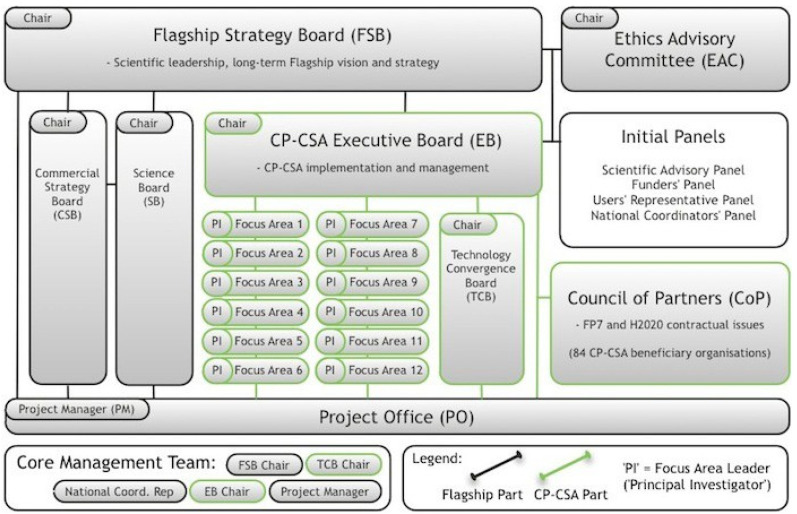
FuturICT Governance diagram (Source: FuturICT Consortium, 2012).

The present paper builds upon a methodological triangulation by combining multiple theories, methods, and empirical materials [23,24,25,26]. First, in order to crystallize information about the FuturICT project, we have conducted a content analysis [27] of published as well as unpublished documents, in order to provide a basic understanding of the (chronology of the) project as well as to distinguish major topics of controversy and to identify key interview partners. Second, one of us (MG)—in the role of an ethnographer —has attended many conferences and workshops that were organized by the FuturICT team, whereas another one of us (DS) has attended some of them while being involved as a focus area leader. Third, 31 interviews were conducted with the project’s consortium as well as its partners and supporters between November 2011 and August 2012. Expert interviews are conducted in empirical social research when information is to be collected that can only be provided by a selected group of people. Thematically, the scope of this information is often narrow and focused on a specific question. Experts are persons who have exclusive knowledge and are usually members of a specific organization or institution, in this case, academia. All interviews were conducted using a semi-structured technique, i.e., using a list of questions asked of each interviewee, added by supplementary questions asked individually [28,29]. The interviewees were to answer freely, they did not have to adhere to any guidelines. Experts were chosen from all levels of the project; this includes three of the six partners of the FuturICT consortium as well as two members of the administration team. All other interview partners were chosen randomly, coming from different disciplines, universities, and countries. The only criterion was that they had to be outside the inner circle of the FuturICT consortium, but still have direct links to the pilot project (see Figure 1). Additionally, one interview has been conducted with a scientist outside FuturICT, but in the same research field.

In what follows, we will start with an outline of the pilot project, followed by a section where we discuss data on how the project was promoted to different stakeholders, namely, to the scientific community, the media, and the public. In the subsequent section, we ask whether the exuberance created by the FuturICT’s consortium was shared by its supporters, and whether, if at all, this mattered. Finally, we examine the risk awareness displayed by the supporters of the project, channeled by our hypothesis that, in the dynamic of a bubble, optimistic expectation leads people to focus almost solely on returns and to forget risks. The last section concludes.

## 2. Outline of the FuturICT Project

In 2009, the European Commission launched an initiative in the field of Future and Emerging Technologies, the so-called FET Flagship Initiative, reaching beyond its usual FET projects. Its ultimate goal was set as to ‘move the ICT frontiers’ further and, hence, to push Europe into a leading position in Information and Communication Technology (ICT). The scientific community was requested to design grand scientific challenges that required a common European research effort; this included major, long-term multidisciplinary activities and visionary research with a high potential pay-off. The projects were expected to result in a transformational impact on science and technology, in order to deliver substantial benefits for European competitiveness. The commission consequently called for a EUR 1 billion over 10 years “Big Science” initiative, with genuinely transformative potential and a “Man on the Moon” scope of vision. Such high-risk ICT research was expected to allow major public and private investments instead of short-term market-driven research priorities.

Initially, 21 pilot projects entered the FET Flagship competition. After a first round, these were reduced to six projects. The final two winners were publicly announced on 28 January 2013 (http://cordis.europa.eu/fp7/ict/programme/fet/flagship (accessed on 6 December 2015)).

FuturICT was one of the six potential flagship topics that were chosen within the European Commission’s Flagship Initiative to be funded over a preparatory phase of one year. The origins of the FuturICT project can be found in the academic history of a professor at ETH Zurich, Dirk Helbing. Constantly interested in interdisciplinary work, he came up with the initial idea of FuturICT, feeling that the questions society is facing were of an interdisciplinary nature (interview with Dirk Helbing, 23 March 2012). He felt that there were not enough experts able to understand complex systems in order to advise decision makers in politics and businesses. His interest was to build up an expertise on complexity systems at a stage (2008) when the financial crisis had just become visible. Coming from physics, he felt it was the right moment to start investing large amounts of time and money into research concerning society at large, just as was the case with major research questions in other areas.

A consortium eventually picked up the ball the EU commission had gotten rolling, creating a project out of Helbing’s initial ideas and around the commission’s requirements. Meanwhile, many scientists had called for a large-scale ICT-based research initiative on techno-social-economic-environmental issues. An organizational concept for the establishment of a knowledge accelerator was being sketched within the EU Support Action VISIONEER (www.visioneer.ethz.ch (accessed on 6 December 2015)). The EU Flagship initiative was the instrument to subsequently materialize this concept.

**Figure 2:**
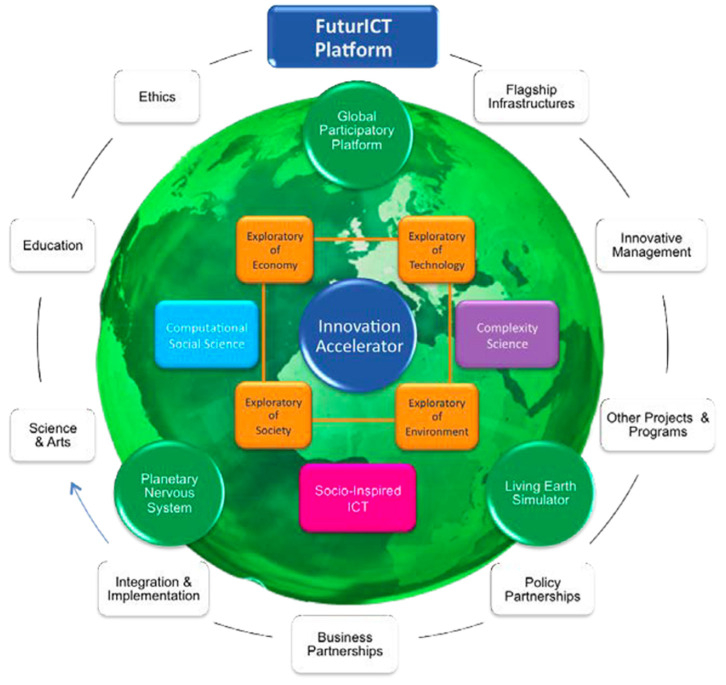
FuturICT platform (Source: FuturICT Consortium 2012).

The consortium’s initial objective was to create an ‘ICT (Information and Communication Technology) Flagship to explore social life on Earth, and everything it relates to.’ The envisioned goal of the project was ‘understanding and managing complex, global, socially interactive systems’ by developing new science as well as new information and communication systems, and promoting social self-organization, wellbeing, sustainability, and resilience. Key to the FuturICT vision was increasing individual opportunities for social, economic, and political participation, combined with the creation of collective awareness of the impact of human actions on the world [30,31,32]. The establishment of ‘a global but decentralized, democratically controlled information platform’ ([31] (see Figure 2) aimed at combining data with models to enable citizens, businesspeople, academics, and policy makers to interact and support future decisions on perplexing societal questions. Methodologically, FuturICT claimed to break new ground in trying to combine three disciplines: ICT, complexity, and social sciences. Its ultimate aim was to ‘shift science.’ Information and communication technologies should be exploited—so the argument went—to achieve major breakthroughs that would reach beyond stepwise developments. This is how the size of the FET flagship was rationalized; for more details, see [33,34,35].

The organization of FuturICT consequently unified hundreds of scholars in Europe and beyond. There were two pilot project chairs: scientific coordinator Dirk Helbing, ETH Zürich, and management coordinator Steven Bishop, UCL, UK. Other partners were Coordination Action Work Package Leaders Paul Lukowicz (German Research Center for Artificial Intelligence, Kaiserslauten, Germany), Rosaria Conte (ISTC-CNR, Rome, Italy), JB McCarthy (University College Cork, Cork, Ireland), and Felix Reed-Tsochas, (Oxford University, Oxford, UK). Furthermore, more than 300 academics from universities from a large number of European countries were involved as focus area and/or work package leaders, respectively, and the pilot project claimed to have nearly 2000 individual supporters who registered to formally back the pilot project [32].

## 3. Promotion of the Pilot FuturICT Project

FuturICT was exceptionally active in presenting itself publicly, either via presentations at events of all kinds or by publishing widely, both scientifically and in social and traditional media (website, Facebook, LinkedIn, Twitter, newspapers, magazines, journals). In fact, according to FuturICT’s own statistics, its members were the top publishers compared to the other flagship competitors.

Yet, communicating the project was not always easy, and it was not always perceived well. The problems started with having to explain something that was going to happen over 10 years—as Dirk Helbing has pointed out. That meant talking about something that would happen in the medium-term future, but with images from the past (interview with Dirk Helbing, 23 March 2012).

In a press release of 28 April 2010, after having submitted its proposal to the European Commission’s Flagship Program, FuturICT presented itself as a future EUR 1 billion EUR project to ‘unleash the power of information for a sustainable future,’ stating that a diverse group of leading scientists had unveiled an extraordinary plan to meet these challenges through a project inspired by historic enterprises such as the Apollo Program. The proposal aimed at building a ‘powerful and accurate science of human systems’ by exploiting the ‘revolutionary scientific potential of modern computational, communication, and information technologies, backed up by theoretical analysis.’ A ‘collective mind’ was targeted, enabled by resources gathered on an ‘unprecedented scale,’ that would be able to make ‘credible and actionable forecasts’ useful to policy makers.

In a paper published in the Cornell-based international electronic archive “Arxiv” http://arxiv.org/ (accessed on 25 September 2021) [36], the FuturICT initiative called for science to catch up with the speed at which new problems and opportunities were arising in a changing world as a consequence of globalization and of technological, demographic, and environmental changes. Its initiative had started as an answer to the rising perception of the serious crises that ‘humankind is facing’ in order to ‘develop new ways to tackle the global challenges of humanity in the 21st century.’ With connectivity between people rapidly increasing, so the argument went, information and communication technologies would be exploitable in order to achieve major breakthroughs that would go beyond stepwise improvements in other areas. Borrowing heavily from physics terminology, FuturICT claimed to use ICT to ‘explore social life on Earth, and everything it relates to,’ in the same way that scientists over the past decades were trying to understand ‘the physical world.’ This meant being visionary, ambitious, and providing novel concepts.

The spirit to ‘address really big problems’ and to ‘answer some of the fundamental questions humanity is facing’ was kept alive during its entire expansion phase. In June 2011, at the official FuturICT Kick-Off Workshops—after being selected as one of the six pilots—the project was presented as seeking to ‘understand and manage complex, global, socially interactive systems, with a focus on sustainability and resilience’ by ‘building an information system that saves the world before the catastrophe is here’ (ethnographic field notes, Kick-Off meeting, June 2011).

The event was organized to discuss the preparation phase of the project’s proposal. Key players of all three disciplines—ICT, complexity, and social sciences—were invited to detail the vision, the research agenda, and the organization of FuturICT. Its core idea was then sketched as constructing a computer model of the world, based on data mining, and eventually conducting experiments to see under what conditions different futures were to play out and to estimate how likely they were. The idea was to mitigate the chances and pathways of crisis situations and potential catastrophic events, such as the global financial crisis, epidemic diseases, generalized crime, and governance failures. The consortium’s goal was to integrate ICT with complexity and social sciences, getting there by ‘developing new scientific approaches and combining these with the best-established methods in areas like multi-scale computer modeling, social supercomputing, large-scale data mining and participatory platforms’ (ethnographic field notes, Kick-Off meeting, June 2011). In bubble rhetoric, one could state that, for marketing reasons, the FuturICT’s consortium created exuberance by overselling the target.

In a book chapter published in June 2012, Helbing seemed to be convinced that, without an initiative of this sort, the world is ‘sure to fall increasingly behind the reach of orderly planning and management’ [37]. He blatantly sketches a project that is seeking to create an ‘open and pluralistic, global but decentralized, democratically controlled information platform that will use online data together with novel theoretical models to achieve a paradigm shift in our understanding of today’s strongly interdependent and complex world and make both our society and global ICT systems more flexible, adaptive, resilient, sustainable, and humane through a participatory approach.’ Helbing acknowledged that this ‘is an ambitious goal, but one that is within our reach.’ Pointing to a democratized system that serves society at large, the project targeted to have technology become ‘more socially adaptive’ and social scientists become more computational, while academics studying complexity science would solve ‘more and more complicated problems that involve both technology and social behaviors’ [38]. In other words, the project aimed at establishing a technology-based democracy, based on an “expertocracy.”

Over time, however, the consortium came to realize that the concept of FuturICT was quite different from putting a man on the moon, mainly due to its lack of a well-defined target and its difficulties in outlining a clear-cut definition of the goal of the project. It sometimes seemed as if the rhetoric blinded people from precisely articulating what it was that the project was supposed to be doing. This made it even more problematic to present it in public, via media and presentations.

Following the blogs and comments of the media coverage, it became obvious that FuturICT was indeed in a position to attract the public’s attention at large, although not exclusively in a supportive way. And in fact, people tended to take the images they already had and project them into the future. In articles and blogs, public commentators compared FuturICT with movies they had seen or books they had read, such as Asimov’s psychohistory *Foundation* series.

The FuturICT consortium reacted to these critics. Over the course of time, it gradually changed its discourse, though never truly its core vision. By shifting from promoting some of its former core concepts, such as the Living Earth simulator, to more democratic ones, particularly the ‘participatory platform’ as its core technology, the consortium eventually accentuated the social and participatory factors of the project in order to feature its democratic intent. Another example is the rhetoric shift from ‘global computing of our complex world’ to ‘participatory computing for our complex world’ (italics added). It was the intention and the strategy to bring the idea behind the project up front to ‘the general audience,’ in order to ‘stand out by integration and access to the public’ (Scientific Advisory Board Meeting, 12 September 2012). Yet, scanning the most recent FuturICT publications revealed that, while the rhetoric changed and the vocabulary was reshaped, its vision and goals remained the same.

Another important dimension of the enthusiasm exhibited by the FuturICT Consortium is revealed by its claims to construct global modeling tools for social sciences, in particular, to develop a ‘‘Policy Simulator’ or ‘Policy Wind Tunnel,’ allowing people to test multiple options in a complex and uncertain world, […] thereby giving politicians and decision makers a better understanding to base their decisions on’ [31]. The so-called ‘Living Earth Simulator’ was proposed to ‘explore questions about our world’s most pressing problems’ [39]. We should note that many in the social science community have raised concerns regarding this program, arguing that FuturICT’s rhetoric was reminiscent of previous failed attempts to develop ‘global modeling’ tools. In this respect, many attempts come to mind, not least the misinterpretations around the well-known and controversially discussed book *The Limits of Growth* [40]; (see however [41]), but also an ambitious project led by Karl Deutsch at WZB in Berlin in the 1980s that failed. In fact, macro-simulation acquired a really bad name in the 1980s and 1990s, especially among social scientists [42]. During the development phase of FuturICT, some social scientists (who were active members) were expressing their concerns that the project would repeat the mistakes the pioneers of social simulation had committed a couple of decades earlier, such as ‘trying to predict the unpredictable’.

Notwithstanding such input, the pilot project successfully created critical mass, building itself as being so important that people felt motivated to participate. In fact, it seems to us that the FuturICT consortium tried to build a system that was “too big to fail” (or perhaps more appropriately “too big to dismiss”) in that it created a community of academics and people from other areas with different scientific and intellectual interests. The ambitious vision and goals of FuturICT worked well initially, creating incentives for scientists to join the pilot project, coming for its sheer attraction. The project ultimately got so visible that many perceived it as central to be part of it, whether for funding reasons, to avoid missing an opportunity, or just to belong to a group of high-profile scientists. Furthermore, the pilot project attracted more than 120 institutions: universities and research centers in almost 25 countries, business companies (e.g., Disney), not-for-profit organizations (such as the Club of Rome), several supercomputing centers, and international organizations such as the World Economic Forum (Email, 5 January 2012).

Going beyond figures, in the following section, we discuss whether or not participants and supporters of the project actually shared the vision and goals promoted by the FuturICT consortium and, if so, to what extent. Our social bubble hypothesis predicts that during the formation of a project, supporters inspired by the very idea of the project are needed, thereby building strong social interactions to push a project further.

## 4. Is the Exuberance Shared by the Supporters—And Does It Matter?

### 4.1. Methodology

The observation of several events (workshops, meetings) exposed the fact that enthusiasm was quite high during gatherings. People involved were generally committed; it was palpable that they clung to the ideas and were ready to support them and to provide expert knowledge to build up the project (but also, in the long run, to get some funding). At the above-mentioned FuturICT Kick-Off Workshop in June 2011, the motivation of the participants was generally high, and a strong responsiveness to the project, as well as strong cooperativeness, was tangible. Many of the participants exhibited a surprising level of enthusiasm, e.g., by expressing a tolerance toward the requirements of the EU commission which, at this point, did not seem to stick to its initial promises concerning the funding. Also, at a later stage of the proposal phase, people enthusiastically contributed to the final draft of the proposal (ethnographic field notes, 4, 5 October 2012).

Yet, since enthusiasm is hardly measurable even in terms of qualitative research, we sought to better understand what made these individuals (mostly academics) join the project. We therefore created a questionnaire trying to capture their commitment to the project as well as the risk awareness among the participants. In the interviews, we asked what made the participants support the project, what their own visions and expectations were, and to what extent they were ready to engage. We have grouped our results into five categories (the consortium, the “visioneers,” the “science-driven,” the “networkers,” and the “critics”), which we will discuss separately (and it goes without saying that the delineation between the groups is somewhat blurred). We have interviewed five people from the consortium, including its administration team. The “visioneers” and the “critics” constitute the majority—roughly seven interviewees each—whereas the other groups, the “networkers” and the “science-driven”, are slightly smaller—six and five, respectively. The main characteristics associated with each group will be accentuated by quotations from the interviews, followed by some general remarks.

### 4.2. The Consortium

It goes without saying that the members of the consortium and the administration were all positive, and confident that the project was unconditionally required and that it was in fact “high time” to pursue it, and that it needed to be done here and now. The project was perceived as being a big step and an important project that would benefit society at large in that it bridges the gap between technology and society: ‘There are completely new possibilities that open up with much fewer constraints than in the past. Now the issue is, how can we use it to the benefit of society?’ (Interviewee 5). It was seen as a high-profile project that was in a position to create a more democratic system through technology (data and model commons) and which would have an impact on science, policy, and technology at large: ‘My angle is more the sort of decision making for people and policymakers, that I would like a little bit more evidence-based policy. [There are] decisions where you could help people understand why those decisions need to be made by actually providing more evidence. So I believe we should be able to do that. And I think in the way that the computer scientists now made me think is that we can open that up to individuals and interest groups much better than we have done in the past. Scientifically, it will give us a much better understanding of how things work, that’s really quite important’ (Interviewee 8). Many interviewees of this group assured us that the project just needed to achieve its first steps, and from there it would spark up and spill over into other areas.

Essentially, the project’s core idea was framed exuberantly via formulation of high expectations in terms of potential outcomes with such great impacts that it could not be ignored: ‘So we are creating a feeling of belonging to a very large community with very challenging outputs in the future—and also that we can have a different impact on society, on research policy, because this will impact the decision of our scientific policymakers, because they cannot ignore the existence of such a community, anymore. So, what to do? Just take us into consideration!’ (Interviewee 2).

### 4.3. The “Visioneers”

This group supported the consortium’s vision categorically. Its followers unequivocally shared the vision that something should be done for society on a large scale and in due time, especially in terms of crisis mitigation in finance, health, and corruption. They also held the view that it was high time to do it, and that it was FuturICT that would be in the position to do it, thanks to its size, its power to attract significant people to join in, and its capability to raise the relevant questions and to acquire the necessary funding.

The interviewees of this group understood the project as a unique, compelling, inspiring mission and one of the leading scientific endeavors in the world. They thought that others would join the bandwagon eventually because the project was seen as pioneering and because of its sheer size. The project was seen as already successful in building a strong community, which would make it effective (‘[…] this amazing collection of experts, who support this common vision and everyone applying a concerted effort to approach this vision’ (Interviewee 30)). The claim by the consortium to shape it as a CERN-like collaborative research was well understood and shared. The management was seen as capable of uniting people coming from different disciplines and trying to work on other people’s ideas with totally unconstrained views. One interview partner was convinced that the project would make people think in a bigger way; in fact, the only potential downside was seen as to not compete at all: ‘In terms of vision, FuturICT is a long way ahead, but very quickly it will have competitors building observatories and exploratories, and so I think there will be a lot of competition, but people realize that if you have a big observatory with lots of data, everyone is going to come to your door, which is also the vision of all the scientists coming to a sort of virtual CERN to do collaborative research’ (Interviewee 11).

The project’s long-term vision was seen as being important, since this would allow people to tackle not only the big questions but also to include marginalized aspects and hence to build a useable architecture to plug in and carry out their own research, in order to provide a lot of scientific knowledge, spinouts, and technology development in the long run.

Moreover, the project was seen as positive not only in terms of science but for society at large. It was understood as being imperative in terms of a better notion of society and its use of information technology, social media, and other information tools, i.e., for closing the society–technology gap and hence preventing, mitigating, and managing crises. It was seen as being unique in integrating different disciplines, especially complexity science, or, even more, in looking at society as a complex system: ‘Economics sees people reacting to incentives, and that’s not wrong, people do, but increasingly the principle motivation is copying or imitating, and networks are the driving force of a great deal of behavior, and so I’m sure scientifically this vision of the economy and society as connected complex systems is a major step forward, it is an intellectual revolution; so I’m very much in tune with the vision of FuturICT’ (Interviewee 21).

‘FuturICT will help solve real world problems’ was a general stance among the “visioneers.” In this sense, the exuberance that was created was understood as being essential. Scientists need to raise promises to society, as one interviewee stressed; the project thus needed to be sold in that way. Yet, one has to be careful, another one pondered—not to promise too much and to oversell the whole idea. Consequently, it would be important to create and sell visions but to also show some reservation, being honest that not all promises would be achieved in due time. Yet, another stance was that the project needed to create critical mass and hence to become so important that people were just driven to join in.

Difficulties were seen not in terms of size or people but in terms of integrating or networking with certain influential groups, such as policymakers, and also with trained social scientists. One interviewee pointed to the issue that the project might move too slowly, so that it might be overtaken by other countries (namely the US and/or China), since other (smaller) initiatives would also be in a position to tackle some of the research questions FuturICT was bringing up.

### 4.4. The “Science-Driven”

All of the other interviewees expressed reluctance about the overall vision (the “mission”) of the project in one way or another. They assessed the achievable objectives of the project at a much lower level. Scientists from the “science-driven” group expected the project to push either science or technology forward, but no more than that, and they were reluctant to determine whether this would yield any gain for society and, if so, to what extent. Most were nevertheless interested because they were optimistic that the project would drive their own research agenda forward, or—in other words—they were hoping to push the core ideas of the project in their very own research direction.

They consequently agreed upon some of the challenging research questions that FuturICT had launched, namely, how to bring big data to models and how to involve stakeholders and decision makers into modeling: ‘There is the big vision of creating these simulators and making predictions; but I think that, at the lower level, there has been more concrete things. […] The big vision, which I may interpret differently than others, for me, it’s building platforms or tools that scientists can use to do big data experiments. That’s the part of the vision I share, there might be other visions that are more ambitious that I might not share as much’ (Interviewee 27). They were keen and in the position to contribute their expertise to these questions. As a reward, they expected to push their own research agendas forward. One of the interviewees stated that s/he would strive to achieve advancement in her/his discipline, and was optimistic that FuturICT would generate research opportunities. This would help increase the quality of research, helping to establish Europe in the field. Hence, s/he felt that it was important to be part of the game, even though s/he did not necessarily share its overall goals.

Another one agreed that FuturICT was very much compatible with her/his own research agenda (big data science; risk/resilience and complexity science) and s/he was interested in pushing those subject matters further within FuturICT. S/he was convinced that some of the expected outcomes would take longer to obtain in the absence of the project, and expected the benefits to be significant. One big project instead of many small ones was seen as intriguing, in this respect.

The overall umbrella of the project was meant to provide possibilities for working with “Big Data”, which would open up new opportunities in many research fields. Interviewees agreed that the size of FuturICT would have helped make sure that these data were acquirable. Many were attracted to the idea of big data becoming a public good, allowing some of the really big questions to be pushed forward. One interviewee showed optimism that whole new businesses and services would build up on top of these data and felt privileged to be a part of it. Another one disapproved vigorously with the overarching goals but shared the prospect of building platforms and tools that scientists could use to carry out big data experiments (even though *not* predicting the future, as was occasionally implied). S/he expected big data research to turn into a revolution, and would be happy for FuturICT to participate. Furthermore, s/he expected FuturICT to succeed in revolutionizing segments in ICT. In fact, only one interviewee expressed great concern in this respect. Even though the sheer size of the project would have made it possible to acquire some data, s/he pondered, the project would nonetheless have problems adding much to what is already known in her/his own research field: ‘There is the question, can we get access to the private observatories, which are there […]; and I am afraid they won’t give us access anyway, because they are using these data commercially; so I’m afraid they won’t give us independent and free access to all the data they have’ (Interviewee 3).

### 4.5. The “Networkers”

Members of this group were very much in favor of being part of the network that was being created in the course of the development of the project, finding it both advantageous and fun. They appreciated the opportunity to work with numerous experts from their own field and, even more so, from other disciplines. Thanks to its size and multidisciplinarity, the project was seen as being in a position to integrate different mindsets, ideas, and ways of thinking. A project as important and famous as FuturICT, built on the expertise of many scholars involved, should later be all the more able to attract stakeholders essential to the project: ‘It’s good to create a network, to create awareness, to align the activities. […] I think it’s interesting to be part of a large network […]; it’s a marketing thing towards our industry customers’ (Interviewee 4). It seems that one has to be in a very secure position to be oblivious to it and to simply state that ‘I do not need it [the network]’ (Interviewees 14 and 31). Bringing together people from different scientific communities, and learning from them, were seen as added values to other opportunities the project was going to provide (‘the value is in the people’). Anyone in this group felt that the project was already outstandingly successful in terms of network building.

Only one interviewee identified a caveat in terms of the network: ‘No, I trust this network, but I am a little bit worried they overemphasize the networking, not the real thing. Networking makes you famous, this person knows everyone else, but does that increase the research of that person? It’s still the same person. […] As any scientist, I would like to be in that cycle, in that networking, because it is very important. But that is the danger of it as well. For example, so, this is FuturICT, they have a bunch of scientists or famous people in FuturICT, does this mean that anyone not in FuturICT is not good? That is the danger, the danger of a big project because everyone says you should be there, why aren’t you there? That is the thing that worries me in that sense. This is the danger of a big project; you have to be very careful about that’ (Interviewee 26). Furthermore s/he pointed to the difficulty that any network that is getting too big is at risk of becoming unstable, eventually crashing due to over-connectivity.

Most people involved—and not only from the “networkers’” group—did not see it this way, though. On the contrary, the effort towards building a huge network has borne fruit. People did not need to agree upon the overall goals to nevertheless want to join the network, hence becoming an insider. Many felt that this was by far the best thing FuturICT had produced at all, all the more since the debate kept circling around networks of all kinds. It seemed that creating its own network was a logical consequence: ‘Particularly, in a project that studies networks, using a network structure as an organization principle seems not to be the worst of ideas’ (Interviewee 6).

### 4.6. The “Critics”

This group embraced those who were critical in terms of the project. They did not share the overall vision, nor would they have believed it to be achievable. On the contrary, they maintained that not only were some of the promises very badly informed, but some of the fundamental claims were completely out of range.

Even though the project was seen as an interesting and courageous experiment, and people found it worthwhile to observe where the idea would lead to—so one interview partner rationalized—its promises (‘We are measuring everything, networking everyone, and then we are going to solve everything’) were understood as utterly naive. The illusion of a new era that was about to happen was really only an illusion, s/he insisted. Furthermore, gains from involvement of her/his own group were seen as zero, since they were in the position to move at a much faster pace, thanks to a smaller size and better flexibility. The “critics” thus teetered on the brink of the project. Nevertheless, they felt they needed to be there after all, since the project had thus far been constructed as inescapable: ‘I’m making a bet that somehow the project has become inevitable, the managing directors made it that, if you are not in, it is like you miss the wave of the time, the big event’ (Interviewee 14). Using buzzwords is even counterproductive—another interviewee assessed—since they promise too much. This would raise a lot of fear unnecessarily, and might backfire in the end, a situation that would need a long time to recover.

Most people in this group felt that the goals the consortium claimed to achieve were highly oversold, too complex, and utopian, ‘ridiculously over ambitious,’ and would never be accomplished: ‘I think humanity’s track record of using technology for good is pretty poor. So unless something happens between now and FuturICT, I think, you know, there is a possibility that it might be used in a negative way’ (Interviewee 15). A comparable criticism from one interviewee was that the concepts of social sciences were not sufficiently integrated and understood, even though this project was supposed to be about society at large. The claim to ‘save the world’ from all its problems, with just enough computing power and advanced models, lacked an understanding of the types of challenges that were involved, namely in terms of social improvement, managing social relations, and getting into politics. S/he further added that, ‘There is on the whole too much obsession with large amounts of data, large amounts of computational capacity, technocratic solutions, where in reality the real problems are conceptual and theoretical. So, no amount of supercomputers would really solve the problems if you don’t have the right theoretical and conceptual ideas from the beginning’ (Interviewee 28). Yet, s/he believed that there was a potential that something useful would come out at last, which was the reason for her/him to stay on board as a ‘skeptical member’ of the community. The interviewee thus adopted a wait-and-see attitude—a stance on the project that was shared by others. Some in fact adopted a cost–benefit attitude that translated into waiting to see what was going to happen and what would be the best moment to jump on the bandwagon. A junior scholar, when asked what that meant career-wise, assured us that it was important to be part of FuturICT, to remain sympathetic, if only ‘in a low priority.’ (Interviewee 18).

### 4.7. Synthesis

Generally, people felt that this project meant a big step forward, even though it remained unclear where it was heading. Many felt it was courageous and were keen to observe where it was going. Others could not share the rhetoric the consortium was adopting, and yet agreed upon the direction of the project. Overall, most did not share its optimistic stance, the enthusiasm the management was so avidly implementing in order to promote the project. Yet, even those who heavily criticized the project were still there, and it was interesting to understand why. One obvious motive for staying on board was that people found niches for themselves within the pilot project, where they felt they could contribute as well as benefit in one way or another, hoping that FuturICT would deliver sooner or later. They expected FuturICT to open up new opportunities, in terms of their own research (in particular for funding), or in the broadening and optimization of their respective network, that might have borne fruit in the future. Most felt they could gain from the fact that FuturICT was supposed to be a multidisciplinary project that might be in a position to shape or further science or technology in general, or at least their own research field. Another important reason for joining and staying with the pilot project was the need to tackle some of the outstanding themes framed by FuturICT. Many interviewees believed these questions would be approached anyway in the near future, so it would be better for FuturICT to tackle them, and far better if they were part of it. After all, the various degrees of interest in the project resulted in an eagerness to somehow invest into the project, either immediately or later on—be it time, money, or reputation.

## 5. Risk Awareness

### 5.1. Overview

Our social bubble hypothesis claims that the dynamics of a social bubble develop by participants taking inordinate risks not justified by a standard cost–benefit analysis. So far, we have come to realize that people did not necessarily need to share the goals that underpin the vision of the overall project. Even though some did, others were reluctant and kept to more modest goals and purposes. In alignment with our hypothesis, we asked our interviewees (indirectly) about potential factors associated with the project. We were interested in examining how the results of the previous sections would be reflected in the answers regarding our hypothesis. More precisely, we were interested in understanding how people saw the risks of the FuturICT project at large and how they assessed the risks of being associated with it. Moreover, we were interested in examining how much they would be ready to invest into the project, in terms of their time, reputation and/or money, on the basis that people who share the excitement for a project should be ready to engage in investments of all kinds.

When referring to risks, we need to distinguish between two types of risks that participants of FuturICT had to face: (i) the risk of the project not being financed (as finally materialized) and (ii) the risk of not meeting the declared ambitious goals once the project started being funded (and hence risk (i) would not occur). Both risks, and more precisely the absence of their perception or the downplaying of their importance, are potentially useful signatures of a nascent social bubble. The first risk was found in general to have a relatively minor role in the minds of the participants; the major risk identified in our interviews was the second one.

### 5.2. Expectations Revised

The most astonishing finding was that the majority of the interviewees (more than 60%) across all categories (except the consortium) stated that the project would by no means deliver what it was promising. The assertion was that it would produce some results, but not what was promised in terms of outcomes, products, or ideas. Out of this 60%, half of it considered it “normal” that this was the case. Hence, there was confidence that something useful would come out eventually—if not scientifically, then intellectually or with respect to networking, providing new opportunities in the future. One interviewee (from the “visioneers” group) put it as follows: ‘Things often don’t turn out as expected, that is rather the norm; […] it’s part of the design’ (Interviewee 30); one of the “networkers” group supported this stance, stating that ‘We can have some ideas, some will be realized and others won’t, and then some things we don’t even think they could come will develop’ (Interviewee 6). And even someone from the “critics” group showed no concern in this respect: ‘This is a risk with every research project, you invest a huge amount of time and effort and you have no guarantee of its success’ (Interviewee 9). The unknown as well as potential failures were understood as being part of the scientific process [43].

This did not mean though that people did not see any risk whatsoever. On the contrary, they showed remarkable risk awareness, calling upon many different factors: ‘Yeah, the time scale means it’s risky. The interdisciplinary thing means it’s risky, the size of it means it’s risky. I think there are quite a lot of risks’ (Interviewee 20).

There is little surprise in finding that the consortium and the “visioneers” expressed the lowest risk level, whereas all others saw medium to high risks for the project. But this was not unequivocal. Whereas one interviewee from the consortium saw potential risk in terms of the project’s size, in the “science-driven” group, two interviewees did not see any risk for the project. In the “networkers” and the “critics” groups, we found similar results, but, even there, not everyone perceived the project as very risky. This again had to do with what position they took in terms of claims and expected results—but there were other factors, too, that were coming into play. Altogether, there was a relationship between the categories and the level of their risk perception (i.e., the more someone agreed with the claims and promises of the project, the less risky the project was perceived, and vice versa), but the differences were rather significant, and it is worthwhile looking at them in detail.

### 5.3. Risk Inherence in Science

The one risk factor that was mentioned most often, by far, was that the size of the project would jeopardize the endeavor. The project was perceived as getting too big, in particular with respect to the request of the EU commission to qualify as a “Big Science” project [44]. The overall concern was that, at some point, the project would no longer be manageable and controllable and that the structures were going to be too fragile: ‘And it’s really up to a small number of people to call the shots, and decide how to do this in terms of rule making, regulation, management structure. Well, again, I’m not convinced that this will be able to carry the weight of the overall volume of research and resources invested’ (Interviewee 28). Furthermore, the apprehension was that, with too many people involved, the communication flow would not be guaranteed, hence destabilizing the network. At the same time, one interview partner emphasized that, if all these frailties were going to be addressed properly and in time, huge gains were to be expected. Other factors that were mentioned at least twice were: time constraints; too much administration requested by the EU; lack of a clear definition of the project (or, in other words, the lack of a “gadget” as an end product); ideas that have not been thought through thoroughly; lots of rhetoric, yet too difficult to grasp what the project is about; lack of success criteria/of quality control; fear that it would move too slowly or that its core ideas will be overtaken eventually by other countries and/or private companies; fear that it was too academic driven and would hence be unable to attract industry.

Interestingly enough, one interviewee reflected only upon a successful termination of the project and what would happen then: ‘Let’s say if you are successful in predicting a crisis, that would be a tremendous change of the way politics works. I mean what is then the role of politics; they are just then kind of the executor of what the crises observatory tells? The whole deliberative way and the reflective structure, at least partly, that would change quite dramatically, and so you have to take that into account. That’s why I think it doesn’t work, because the people will then anticipate what is going on’ (Interviewee 7). Another one brought up the problem of how to break up the three disciplines involved: ‘Risks: that the scientists will never quite break down their disciplinary silos; that like in some European projects on a small scale, people come and give up trying to work together and just spinoff into their little activities’ (Interviewee 24).

On the other hand, many pointed to the importance of risky projects in science. Individuals needed to explore to satisfy a demand for achievement, many interviewees maintained—to do something special, often out of pure scientific curiosity. Funding science is not comparable to building a factory, one interviewee pointed out; science has to be inherently risky: ‘Whatever we do has always failure built in; otherwise it is not interesting from a research perspective.’ Failure in that respect was seen as ‘part of the program,’ where one starts to learn. The difference from failure in industry is that one is not necessarily losing one’s job just because a project does not turn out as expected: ‘If we fail at what we do […], then we say, that is life, that is research’ (Interviewee 27).

One could counter that scientists do not care because it is not their own money that is at stake here. Yet the interviews revealed that many were fully aware and pretty careful with spending taxpayers’ money. Furthermore, if it is not their money, then it is certainly their reputation that may be jeopardized: ‘You were asking about reputation: I won’t lose my job […] unless I do something really crazy—this is my personal thing to not lose my job—but I can lose a big part of my reputation. In research, you don’t lose reputation because you do projects that fail, in research you lose reputation because you miscommunicate, you raise expectations, you conduct poorly. […] As researchers, it is our task to take risks, we are supposed to do things others cannot do’ (Interviewee 27).

The risk perceived was not concerned with an unknown outcome; this was seen as ‘science as usual.’ On the contrary, several interviewees urged the provision to people of opportunities that include trial-and-error, and not to punish them if they failed. Because otherwise, most agreed, one would do only safe things that would not turn into great innovations. From our social bubbles perspective, we should add that, even if projects fail in the short run, they might still deliver outcomes in the long run, as demonstrated by previous case studies [1,2,3].

### 5.4. Investment Assessment

One would expect that the more one shares the vision of a project, the more one is ready to invest into it. Our questions therefore aimed at revealing whether people wanted to invest into a project they “believed” in. Yet, this was not exactly what came out in our analysis. We found that one does not need to share the overall vision in order to be ready to invest in a project, be it time-, reputation-, or moneywise. In fact, only the “critics” showed little interest in investing fully in the project; all other categories revealed relatively equal interests to invest, independent of their concern for the vision of the project.

It goes without saying that all interviewees were ready to invest some of their time into the project; they would not even see it as a loss, should the project not succeed as expected. In terms of investing money, it became evident that most expected to get some funding back sooner or later, even though not necessarily much, since the slices of the pie to be distributed among the many participants were expected to be quite small. Not many, though, felt that they wanted to provide capital, unless it would be in terms of time or in opportunity costs. One of the “networkers” replied, when asked for potential funding: ‘At the moment, no. This is risk capital, it’s too risky, too early. I already invested my time, this is capital. To a large part, it’s overtime, so if I compute a value, it’s several tens of thousands of francs’ (Interviewee 4). Others shared this view in that they confirmed that they already had invested quite some capital in time value. A rough quantitative analysis suggests that at least 50% of the interviewees (consortium and administration not included) invested at least 20% of their time at a point where funding was not guaranteed. Many were ready to resume this investment once the project took off. As mentioned above, many also expected to get some funding in due time. It was only the “critics” group that declared an intent to invest as little time as possible or, at any rate, to wait first and decide later whether to devote more hours.

On the basis that scholars with a high reputation co-determine what direction a project takes [45,46,47], we were curious to understand how much of their respective reputations they were ready to invest into the project. We obtained somewhat ambiguous answers in this regard, not least because people could not exactly figure out how to answer the question: ‘I wouldn’t even know how to answer that question. I couldn’t quantify it. Reputation is a weird thing to invest; you give your name, yes. If things work out, you have a bigger name, if not, you have a smaller name’ (Interviewee 27). The aspect of investing reputation is not to be underestimated, though. We assume, and our former studies have indicated, that major projects striving to establish in new fields are heavily dependent on people with high reputations engaging in the project, in order to attract other people to come on board.

## 6. Discussion

Has individual risk perception been affected by network reinforcement or collective herding? This is hard to say, given the observed heterogeneity of attitudes. The hypothesis that, with over-optimistic expectations, people focus almost exclusively on the expected returns and tend to forget the risks was certainly confirmed for the members of the FuturICT consortium; with the other interviewees, this was not necessarily the case. As we have shown, the consortium heavily pushed for reaching a critical mass that included a large scientific community. The ambitious vision and goals of FuturICT outlined by the consortium worked well initially, but could not hold up under more thorough scrutiny. The community eventually departed from the ultimate goals of the project. The interviews elucidated that, in order to endorse a project, people do not necessarily need to share its overall goals and visions. In fact, whereas some of the interviewees enthusiastically supported the project’s goals, the majority did not quite do so, and some rejected them entirely. For example, many expressed their concerns about leaving the issues of the information age to private companies, deliberately wanting to engage on the subject. Yet, this did not turn them into ungrudging supporters, sharing the overall vision indisputably. On the contrary, many seriously doubted that the project would meet its great promises. More important to them was to find niches for themselves, something that they found stimulating and fascinating enough to want to contribute, keeping up the spirit of the very idea of the project. The participants had their own ideas regarding the project and showed diverse and individual motivations as to why they participated. As a consequence, they negotiated their own arrangements regarding the project and conveyed rather heterogeneous resources.

At the individual level, our interviews made it clear that the participants showed considerable risk awareness, based on a force of habit, being used to acting in a risky research framework on a daily basis. In this regard, the effort to build a level of exuberance that would put risks behind expected gains has generally failed. Prima facie, this would seem to be in contradiction with the social bubble hypothesis that risks are minimized in favor of a focus on potential gains. However, we need to mention that the present study is the first one, to our knowledge, that goes beyond an ex-post interpretation of behaviors to expose the ex-ante intentions of participants. As such, it may be associated with the well-documented “intention–behavior” inconsistency or gap, according to which people’s intentions are rather weakly correlated with their subsequent actions [48,49]. Indeed, the evidence for risk awareness contrasts with the result that many interviewees expressed their readiness to invest their time into the project, notwithstanding the uncertainty of whether the project would get funded. While individuals stated their risk-aversion, they did exhibit a lack of risk aversion in their revealed actions in terms of time spent. Observations during the ethnographic field search allow us to assert that people were heavily involved, and not only in matters of time investment; their engagement and readiness to throw themselves enthusiastically into the project were almost palpable. Optimistic expectations combined with impatience and excitement were utterly perceptible; revealed risk aversion seemed to be small or even absent. This suggests that we dealt here with a phenomenon where people were risk-aware at an individual level, whereas, when enthused by a group effect, they were ready to invest and act more than what would perhaps be rationalized by a standard cost–benefit analysis. Moreover, given the ubiquitous and well-accepted nature of the risks associated with scientific research and of the FuturICT project, the absence of strong emotional components in the risks may have made the absence of any significant impact on revealed actions likely [50]. The enthusiasm shown by participants during workshops and collective brain-storming sessions could also be reinforced by group thinking [51] and herding behavior, as time is a more liquid commodity to harness—especially in a project that promoted a mixture of top-down and bottom-up organization, mediated by many meetings and workshops. This would result in the conclusion that individual agent behavior is not a good description of the behavior of groups. A possible reading can therefore be that the individual remains risk-aware during social bubbles but acts “as if” risks are forgotten when in a group, due to a herding effect, resulting from collective enthusiasm. In sum, the social bubble develops through the collective behavior of interacting participants. Hence, from the group perspective, our study qualifies the FuturICT project as a social bubble.

Did the perspectives of individual participants in exhibiting clear risk awareness together with the exuberant dynamics of the FuturICT group influence the decision of the European Commission to announce the rejection of the FuturICT project in January 2013? We can only speculate. Let us note that the two selected flagships, Graphene and the Human Brain Project, are much more anchored in the physical and biological sciences. A dimension not touched so far—and which was certainly relevant for shaping the decision of the European Commission—is the political context, and in particular the lobbying dimension in the race between the six projects vying for the final two selections. It is unclear to what extent the outcome of the process can be attributed to “endogenous” or “exogenous” factors, and in particular to the relative strength of bottom-up endogenously generated visibility versus top-down exogenously driven lobbying. In other words, was the initial success the power of the crowd or the influence of a few well-connected individuals? It is very difficult to draw conclusions about this at present, given the available data, but the lobbying dimension will need to be further investigated as our previous studies have documented its important role in the formation of a social bubble.

## Data Availability

Not applicable.
